# CHD2-Related CNS Pathologies

**DOI:** 10.3390/ijms22020588

**Published:** 2021-01-08

**Authors:** Marc-Michel Wilson, David C. Henshall, Susan M. Byrne, Gary P. Brennan

**Affiliations:** 1 Department of Physiology and Medical Physics, RCSI, University of Medicine and Health Sciences, Dublin 02, Ireland; MarcMichelWilson@rcsi.ie (M.-M.W.); dhenshall@rcsi.ie (D.C.H.); 2 FutureNeuro SFI Research Centre, RCSI, University of Medicine and Health Sciences, Dublin D02 YN77, Ireland; suabyrne@rcsi.ie; 3 Department of Paediatrics, RCSI, University of Medicine and Health Sciences, Dublin 02, Ireland; 4 Department of Paediatric Neurology, Our Ladies Children’s Hospital Crumlin, Dublin 12, Ireland; 5 UCD School of Biomolecular and Biomedical Science, UCD Conway Institute, University College Dublin, Belfield, Dublin 04, Ireland

**Keywords:** CHD2, developmental epileptic encephalopathy, epigenetics

## Abstract

Epileptic encephalopathies (EE) are severe epilepsy syndromes characterized by multiple seizure types, developmental delay and even regression. This class of disorders are increasingly being identified as resulting from de novo genetic mutations including many identified mutations in the family of chromodomain helicase DNA binding (CHD) proteins. In particular, several de novo pathogenic mutations have been identified in the gene encoding chromodomain helicase DNA binding protein 2 (CHD2), a member of the sucrose nonfermenting (SNF-2) protein family of epigenetic regulators. These mutations in the CHD2 gene are causative of early onset epileptic encephalopathy, abnormal brain function, and intellectual disability. Our understanding of the mechanisms by which modification or loss of CHD2 cause this condition remains poorly understood. Here, we review what is known and still to be elucidated as regards the structure and function of CHD2 and how its dysregulation leads to a highly variable range of phenotypic presentations.

## 1. Introduction

Chromatin arrangement is a major functional aspect of transcriptional control and thus of the complex co-ordination of all body functions and systems in eukaryotes [1]. Chromatin remodeling proteins constitute a large group of regulatory proteins which modulate the chromatin architecture and regulate temporal and spatial gene expression. They are unified by their capacity for coupling ATP hydrolysis to DNA conformational changes, thereby imparting direct control over RNA polymerase-mediated transcription [1,2,3,4]. There are four major families of chromatin modelers, based on structural differences that underpin functionality in their context-dependent molecular mechanisms [5]: these include the switch/sucrose non-fermenting (SWI/SNF), the imitation switch (ISWI), the inositol 80 (INO80), and the chromodomain helicase DNA binding (CHD) families of chromatin remodeling proteins [6,7]. Several excellent reviews of the chromatin remodelers, their structures, and their overall functions are available [5,8,9,10]. The CHD family proteins are structurally and functionally linked to the other three remodeling families by virtue of their SNF2-like ATPase/helicase domain, but are distinguished within this larger group by uniquely possessing a pair of tandem chromatin organization modifier domains (chromodomains) [11,12]. Dysregulation or disruption of CHD family proteins is linked to numerous diseases, including developmental disturbances and many cancers (outlined below) [13,14,15]. Recently, variants in the gene encoding the chromodomain helicase DNA binding protein 2 (CHD2) were identified as a cause of a rare developmental epileptic encephalopathy (DEE) [16,17,18,19,20,21]. In the current review, we discuss how the canonical role of CHD2 may influence the variable phenotypic presentations of CHD2-developmental epileptic encephalopathy (CHD2-DEE), and the possibilities for future therapies to treat this complex disorder.

## 2. Structures, Functions, and Dysregulation of the CHD Family Proteins

### 2.1. The CHD Protein Family

In 1993 Delmas et al. reported the discovery of a DNA-binding protein that contains both a chromo (chromatin organization modifier) domain and a SWI/SNF helicase domain that is present in most mammals. They named this protein chromodomain-helicase-DNA-binding protein (CHD-1) [11]. Further analysis of related proteins ensued, giving rise to the characterization and description of CHD2 by Woodage and colleagues [12]. It has since been determined that the CHD protein family is conserved throughout the eukaryotic kingdom with different numbers of variants found in each of a vast assortment of genera, including one CHD protein in saccharomyces, four in drosophilae, and nine in vertebrates, including humans [8,9,22,23,24]. Among the CHD proteins, in addition to the characteristic pair of chromodomains and the ATPase dependent SNF2-like helicase domain, each of the individual members has a range of additional domains, each of these imparting various functional capacities [5,8,9].

### 2.2. CHD Proteins; Distinctions and Commonalities

**Figure 1:**
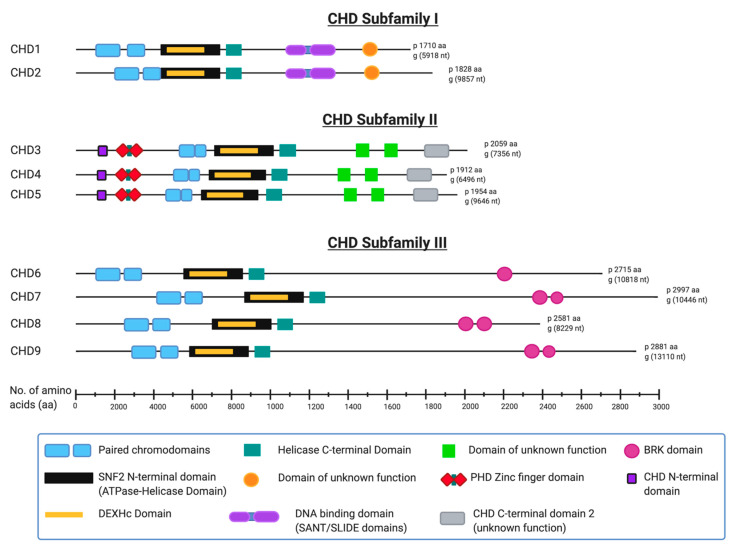
Domain composition of human CHD proteins. The 9 known human chromodomain helicase DNA binding (CHD) proteins, divided into their appropriate subfamilies and drawn approximately to scale with all known or predicted domains included. All structural information was derived from Uniprot [32], InterPro [33], PFAM [34] and the National Center for Biotechnology Information (NCBI) [35] web sites. (Image created in BioRender^®^ [36]).

The array of auxiliary functional domains within each of nine human CHD family protein members underlies their division into three subfamilies [8,9]. Notably, each CHD protein has slight structural differences within its characteristic paired chromodomains, and these compositional variances characterize each member, and therefore each subfamily [25] (Figure 1). The structurally similar CHD1 and CHD2 constitute subfamily I—both having a relatively well conserved C-terminal DNA binding domain [12,26]. CHD3, CHD4 and CHD5 make up subfamily II—having two plant homeodomain (PHD) zinc finger domains [12,27,28]. In contrast to subfamily I, CHD3 and CHD4 function in larger multiprotein complexes and they have no established C-terminal DNA binding domains [10]. Despite its PHD domain and structural similarity to CHD3 and CHD4, CHD5 is sometimes classified as a member of CHD subfamily III, which is comprised of the remaining structurally variable CHD6 to CHD9. This anomaly relates to CHD6 being discovered before CHD5 and its subsequent renaming following the discovery and sequence analyses of CHD5 [29,30,31]. Members of subfamily III generally are classified as such by exclusion from the other two subfamilies, but most have either a terminal conserved hairpin (TCH) motif and/or a SANT domain [8], and a paired Brahma Kismet (BRK) domain [14].

The structures shown in Figure 1 are based on published protein sequence and function information available from protein databases Uniprot, InterPro and Pfam ad from the National Center for Biotechnology Information (NCBI) [32,33,34,35]. Several individual domains of human CHD proteins have been analyzed and the protein crystal structures published including the entire CHD4 structure [37]. The structure of many of the individual domains including zinc finger domains, chromodomains and SNF2-like helicase domains can be found at the Research Collaboratory for Structural Bioinformatics Protein Data Bank (RCSB PDB) [38].

While the highlighted structural similarities and differences between the CHD subfamilies and individual protein motifs are well documented, some uncharacterized domains still remain, whose functions are yet to be established [39,40].

### 2.3. Functions of CHD Family Proteins

The canonical function of CHD proteins is the spatial and temporal regulation of gene expression via reversible structural and chemical changes to DNA to modulate accessibility of the transcriptional machinery [11,41,42,43]. Their tandem chromodomains underpin a major functional attribute of this family in its capacity for recognizing and binding specific, epigenetically labelled, regions of DNA.

Histone H3 lysine 4 di- and tri-methylation (H3K4me2/3) are epigenetic markings associated with transcriptionally active euchromatin [44,45]. While some regulatory complexes and proteins such as the polycomb repressive complex (PRC), and heterochromatin protein 1 (HP1), have single chromodomains that recognize specific lysine methylated histone tails, it has been demonstrated that CHD1 recognizes and interacts with H3K4me2/3 marked chromatin to inhibit binding of other regulatory units, while directing H3 binding to a position between the paired chromodomains [46]. In yeast this activity is further reinforced by the co-operative interaction of Chd1 with the Isw1b complex (which recognizes H3K36) to maintain euchromatin stability during RNA polymerase II (RNAPII) activity [47].

The C-terminal DNA binding domains (DBD) that are conserved in CHD1 and CHD2, bind AT-rich regions of DNA in vitro, but DNA binding in vivo actually occurs via the tandem chromodomains at the recognized H3K4me2/3 sites [10,46,48]. This correlates with the observed DNA binding and epigenetic recruitment activity performed by chromodomains in other protein families. More importantly, however, it suggests that the physiological action of the DBDs probably supports additional distinct functions. Although subfamily II CHD proteins lack defined DBDs, the DNA interactions of CHD3 and CHD4 orthologs in *Drosophila melanogaster* also take place at sites of active transcription and are similarly facilitated by their chromodomains, but H3K4me sites are not necessary [49,50]. In fact, CHD3 and CHD4 are also known as Mi-2 alpha and Mi-2 beta because of their inclusion in the Mi-2/nucleosome remodeling and deacetylase (NuRD) complex which couples ATPase-driven nucleosome remodeling and histone deacytelation [28]. As suggested previously, Chd1 in yeast also functions as the ATPase in larger multiprotein complexes which facilitates epigenetic changes at the histone level [10,51], and there is also strong evidence for a CHD7 and CHD8 containing complex in humans [52,53]. CHD5 is primarily expressed in the brain and testis and is involved in the regulation of transcription in development and of neuronal processes [29,54]. Mice deficient in CHD5 display strong autism spectrum disorder-like behaviors [55]. CHD6 is associated with housekeeping functions, associating directly with various transcription factors including Nrf2, and localizing exclusively in the nucleus where it associates with phosphorylated forms of RNA polymerase II (RNAPII), suggesting involvement in the regulation of a specific subset of genes, rather than as a universal regulator of transcription [23]. This is supported by the suggested function of the SANT domains (harbored by both CHD6 and CHD9), which generally associate with unmodified histone tails [14], as opposed to specific methylated histone tails like the majority of the other CHD family members. In addition, CHD6 has been shown to associate with sites of both active and inactive chromatin, and although a comprehensive understanding of its overall function is still far from realized, it is a demonstrated key factor involved in the response to DNA damage in oxidative stress in humans [56]. CHD2, CHD3, CHD4 and CHD5 have also been implicated in the DNA damage response in various DNA repair pathways [31,57].

In CHARGE (ocular coloboma, congenital heart defects, choanal atresia, retardation of growth and development, genital hypoplasia, and ear anomalies associated with deafness) syndrome (a multiple congenital phenotypically variable syndrome) CHD7 is associated with many but not all instances of the disease [58], and it has been shown to interact both with CHD8 and other proteins in cultured human cells. Structural evidence suggests it may act in a similar capacity to the *Drosophila* kismet protein in interactions with RNAPII [53]. While CHD8 functional data are scarce, dysregulation of the protein is seen in a subset of autism spectrum disorders (ASD) and several distinct developmental abnormalities [52,59,60]. It is also implicated in CHARGE syndrome via its associations with CHD7 [53,59,60]. Indeed, recently a CHD7 binding site has been identified within the structure of CHD8 [52]. Clinical, genetic, and structural evidence suggests that CHD8 plays an important role in the regulation of transcription during early development. CHD9 has been demonstrated to localize and interact in a similar fashion to CHD6 in its association with RNAPII, but unlike CHD6, it does recognize specific methylation marks, H3K9me2/3 and H3K27me3. A variant form of CHD9 that localizes in the nucleolus and associates with RNA polymerase I (RNAPI) has recently been identified [61]. Further, it has been demonstrated that Chd9^−/−^ (knockout) mice are viable and develop normally [62], given the incidence of CHD9 dysregulation in many cancers this finding supports a vital role in the DNA damage response. This also suggests that some of the CHD family proteins may offer some overlap in function and possibly compensation in cases of haploinsufficiency while others may have exceptionally unique functionality. Indeed, several studies have revealed that the CHD proteins can act as co-activators or co-repressors, that they exhibit tissue specific functions, and that recruitment and interaction with DNA occurs at different stages of transcription [8,10,41,63,64].

Taken together, these findings indicate that each CHD family protein regulates transcription by recognizing and binding DNA at specific sites, and interacting with transcription factors, histones, other proteins and/or protein complexes, and very often a specific polymerase. It is also now evident that these interactions, and specific interacting factors, vary for each CHD family member.

### 2.4. Pathogenic Gene Variants in the CHD Family Proteins

Pathogenic gene variants have been described in all members of the CHD family. CHD1–9 have all been implicated in cancers of various forms (Table 1) [13,27,29,54,65,66]. Additionally, CHD2, CHD4, CHD5, CHD7 and CHD8 variants have been implicated in developmental disorders [14,40,58,59].

The table highlights the involvement of CHD proteins in human disease. For comprehensive reviews of CHD family proteins in disease see references [14,72].

## 3. CHD2-Associated Pathologies

While mutations in most of the CHD proteins have been described and demonstrated to lead to a range of cancers and developmental diseases (Table 1) [13,27,40,54,58,59,65,66,71], a number of gene variants in the gene encoding CHD2 were identified as the cause of a developmental epileptic encephalopathy (CHD2-DEE) [16,17,18,19,20,21,73]. This condition is usually characterized by difficult to treat seizures, cognitive regression, intellectual disability (ID) and often autism spectrum disorder (ASD)-like behaviors [74]. Seizures usually develop as early as 6 months old and generally before 4 years of age, with multiple seizure types including myoclonus, myoclonic-absence seizures and drop attacks [73]. Photosensitivity is often present and self-induction of seizures is seen in some patients [20,75]. Several prominent features of the CHD2-DEE phenotype overlap with other DEEs including myoclonic-atonic epilepsy (MAE), Lennox Gastaut and Jeavons syndromes [21]. There is also strong phenotypic overlap with Dravet syndrome, a rare DEE most often caused by mutations in SCN1A. Sensitivity to generalized fever-induced seizures, as well as intellectual disability are common in individuals with mutations in either CHD2 or SCN1A. Indeed, in a study of individuals with Dravet syndrome, one third of individuals who did not have a pathogenic mutation in SCN1A were found to harbor mutations in CHD2 [69].

Aspects of the pathology resulting directly from disruption of the CHD2 protein levels remain unclear and are likely the result of altered expression of CHD2 target genes. The diversity of genes potentially regulated by CHD2 during development likely accounts for the array of phenotypic variation seen in this condition [73,76]. A review focusing on the phenotypic variability of several DEEs recently analyzed the phenotypic variance amongst 56 individuals with a pathogenic CHD2 mutation. While seizures were present in most cases (45/56), other features were more variable and not necessarily correlated with mutation type. About 30% of individuals were also diagnosed with ASD, while 22% of individuals displayed delayed speech. MRI detected several brain structural abnormalities in a small number of individuals, regression was also reported in nine individuals highlighting the phenotypic complexity of CHD2-DEE [77].

### 3.1. Identification of CHD2 as a Novel Epilepsy Gene

In 1991 a report of a previously undescribed chromosome 15 mutation (q26.1 qter deletion) was published in which it was noted that mutations within this region result in variable combinations of intrauterine growth retardation (IUGR), microcephaly, abnormal face and ears, micrognathia, highly arched palate, renal abnormalities, lung hypoplasia, failure to thrive, and developmental delay/mental retardation [78]. Since then, further de novo mutations in this region have been reported, with many of these resulting in varying degrees of early onset DEE, abnormal brain function, and ID (see Table 2). From these initial studies [78,79,80], it was proposed that the resultant growth and neurodevelopmental disruption may be mediated, at least in part, by insufficiency of the insulin-like growth factor 1 receptor (IGF1R) (The gene for IGF1R lies slightly downstream of that encoding CHD2, in the chromosome 15q26 region). However, a functional study into the loss of one copy of IGF1R found that there was no evidence of an impaired response to IGF-1 even when IGF1R expression was decreased in patient fibroblasts [81].

Further reports of 15q26.1 deletions followed and in 2009 Veredice and co-authors published one such report detailing a de novo microdeletion at 15q26.1 in which the patient suffered from refractory myoclonic epilepsy along with minor physical anomalies [82]. Subsequently Dhamija et al. reported an even smaller de novo microdeletion from the same region and described an exceptionally similar phenotypic presentation to that reported in the Veredice paper, thus, narrowing down the location of the disease causing variant [83]. Attempts at identifying a specific causative gene and a characteristic phenotype within this region continued. Then, in 2013, a slew of data published in four seminal papers came together to indicate definitively that the gene primarily responsible for the epileptic component (and thus the aspects of neural developmental disruption directly caused by seizures) emanating from 15q26 disruptions, was *CHD2* [69,73,76,80].

These studies and subsequent follow up analyses firmly established the role of *CHD2* disruption in the majority of cases involving deletions and/or mutation in this narrow chromosomal region, when seizures are present [17,19,21,74,75].

Thus, by 2017 the body of evidence convincingly supported that hemizygous *CHD2* mutation leading to haploinsufficiency causes mild to severe neurodevelopmental disruption in a phenotypically diverse “syndrome” that most commonly results in the presentation of myoclonic epileptic encephalopathy with onset of seizures in the first few years of life, in combination with varying degrees of other cognitive and growth deficits, and dysmorphic features characteristic of disruptions in proximate genetic loci. While this is the case, the mechanism by which epilepsy develops in patients affected by these mutations is still largely unknown, as is a comprehensive and definitive description of the role of CHD2, as distinct from the numerous extant related proteins [39,84].

### 3.2. CHD2 Expression

CHD2 is ubiquitously expressed in all tissue types in humans [12] with low regional specificity including within brain structures and cells [104,105]. The functional outcome of this ubiquitous expression of CHD2 is evidenced by gene variants often presenting non-CNS phenotypes including, other than the morphological, spinal and craniofacial abnormalities already described, monoclonal B-lymphocytosis (MBL) and chronic lymphocytic leukaemia (CLL) [106]. Interrogation of the functional effects leading to these pathologies has shown that when the mutations occur in the region of the DNA-binding domains, association with active chromatin is disrupted. It has thus been postulated that *CHD2* mutation is a driver of cancer, particularly lymphoma [106,107,108].

Recent findings have demonstrated that *Chd2* is expressed prominently in regions undergoing neurogenesis in mouse neural progenitor cells. Specifically, *Chd2* is highly expressed in radial glial (RG) progenitors and is rarely expressed in intermediate progenitors (IPs), which are the primary precursors of neurons, during the development of the cerebral cortex [109]. In young adult (postnatal day 30 (P30)) mouse brains Kim et al. found that *Chd2* is expressed throughout the brain and is particularly strongly expressed in the olfactory bulb, neocortex, hippocampus, and cerebellum. With regard to specific subpopulations of neuronal cells, they found that *Chd2* is expressed in all mature neurons, GABAergic interneurons, and oligodendrocytes but not GFAP-positive astrocytes [110]. Similarly in human cortical interneurons (cINs) derived from human embryonic stem cell (ESC) cultures, Meganathan and colleagues found that *CHD2* expression increased during cIN differentiation [111].

Thus, it has been demonstrated that CHD2 is expressed throughout brain tissues but that differences in expression occur in various cellular subtypes at different stages of development.

### 3.3. Regulation of CHD2 Activity

#### 3.3.1. CHD2 is Transcription-Coupled

CHD1 and CHD2 recruitment is directly coupled to transcription at transcription start sites (TSS) indicating that their regulatory roles are linked to regions of active chromatin [112]. CHD1 and CHD2 have overlapping site specificity and often co-localize but it is likely that they bind with different affinity at those overlapping sites [45,112]. While recruitment of CHD2 to its sites of activity are transcription based (shown to be linked to RNA pol II activity in somatic cells in vitro) and directed partly by methylation of H3K4me2/3 [112], it has also been shown that for high affinity binding of CHD2, supported by the C-terminal DNA binding domain, a region of at least 40bp of target dsDNA is needed [84]. Indeed, the affinity of CHD2 to H3K4me sites has been shown to be lower than that of CHD1 [22] and that CHD2 in mouse embryonic stem cells may function via H3K36me as is seen in yeast Chd1 through its interaction with the Isw1b complex [47,113]. Such observations have given rise to the suggestion that mammalian CHD2 may sometimes function during development analogously to yeast Chd1 [114].

#### 3.3.2. Regulation of CHD2

Early characterization work showed that tissue specific CHD2 splice variants exist [12], implying system-specific functional diversity of the protein. More recently, it has been shown that CHD2 mRNA is a target of, and is modulated in certain tissues by the ubiquitous splicing regulator Rbfox2 [115]. Work by Gehman and colleagues has previously indicated that Rbfox2 is linked to both brain development and motor function and that this function is in part due to interactions with the CHD family protein CHD5 [116]. Thus, following from the demonstrable role of Rbfox2 in neurodevelopment, and the interaction between Rbfox2 and CHD2, it is possible that alternative splicing plays a role in the regulation of CHD2 function in the developing brain. Furthermore, in mouse models of acquired focal temporal lobe epilepsy (TLE) it was found that a microRNA-Rbfox (primarily Rbfox1) interactions controlled synaptic scaling and hyperexcitability [117].

In addition, further investigation into the regulation of *Chd2* has indicated that the long non-coding RNA (lncRNA) LINC01578 or CHD2 Adjacent Suppressive Regulatory RNA (Chaserr), represses *Chd2* gene expression solely in cis, and that the phenotypic consequences of Chaserr loss are rescued when *Chd2* is also perturbed [118]. Little is known about the physiological function/s of this lncRNA although it has been reported that it may have an association with gout [119]. Preliminary sequence alignments indicate predicted conserved sequences in other hominids and Rom et al. indicate that it is a highly conserved mammalian lncRNA [118].

These possible additional intricacies in the gene regulation interactions that govern CHD2 expression and function may account to some extent for the variable phenotypes seen in CHD2 encephalopathies.

### 3.4. The Specific Functions of CHD2

#### 3.4.1. CHD2 Recognizes H3K4me Marks and Deposits Histone Variant H3.3 as an Epigenetic Signal

Due to differences in the chromodomain, CHD2 has a lower affinity for H3K4me than CHD1. It has been found that when CHD2 interacts with sites of active chromatin, it is initially recruited by an RNA polymerase after which it deposits the H3.3 histone variant leading to altered target gene expression levels [22,112]. In some tissues CHD1 and CHD2 can function in a coordinated manner to regulate chromatin organization in several steps [112] showing a clear distinction in function between the two related proteins.

The epigenetic control mediated by CHD2 was uncovered in a study aimed at elucidating how muscle cell differentiation-regulating factors are themselves regulated. Harada and colleagues identified CHD2 as a regulator of MyoD—a transcription factor that determines cell fate. They found that CHD2 incorporates the histone variant H3.3 into genes in cells destined to differentiate into muscle cells. Although such functionality lies outside of the CNS, it provides further insight into a consolidated mechanism of CHD2 activity in development [120].

#### 3.4.2. CHD2 Assembles Nucleosomes into Regularly Spaced Arrays

In 2015, Liu and colleagues, prompted by the implication of *CHD2* deletions in epileptic disorders in humans and developmental defects in both humans and mice, investigated the biochemical activity of CHD2 at the molecular level via a range of biochemical assays. They noted the affinity of CHD2 for chromatin and its capacity for chromatin remodeling, highlighting many of the interdomain control mechanisms discussed in the preceding section. Additionally, the authors sought to determine whether CHD2 plays a role in nucleosome assembly. They found that CHD2 assembles regularly spaced nucleosome arrays from purified components in the presence of ATP. They thus concluded that CHD2 functions not only as a chromatin remodeler in regulation, but also as a chromatin assembly factor [84].

#### 3.4.3. CHD2 Is Involved DNA Damage Repair via Non-Homologous End Joining

Recently, CHD2 has been found to play a critical role in DNA repair mechanisms. Luijsterburg and colleagues found that at sites of DNA double stranded breaks (DSBs), poly(ADP-ribose) polymerase 1 (PARP1) recruits CHD2 to rapidly expand chromatin and deposit H3.3 variants to initiate non-homologous end joining repair (NHEJ) [121]. It was further shown that the C-terminus of CHD2 is the site of the interaction between PARP1 and CHD2. The overall process defines the role of CHD2 as a regulator of stable genetic structure and information [121]. These findings thus strengthen previous suggestions that CHD2 is a tumor suppressor gene [57,106]. Furthermore, it demonstrates that CHD2-mediated epigenetic changes result in different effects, depending on tissue type and on functional context throughout the various stages of development.

#### 3.4.4. CHD2 Regulates the Expression of Developmental Genes

CHD2 has been found to interact with other important developmental transcriptional regulators. Shen et al. demonstrated that CHD2 binds directly to the repressor element 1-silencing transcription factor (*REST)* gene (also known as the neuron-restrictive silencer factor (*NRSF*)). They found that when *CHD2* is silenced, REST expression is decreased. Conversely, when CHD2 was overexpressed REST expression increased. They demonstrated that regulation of neural differentiation mediated by REST is promoted by CHD2 expression, and importantly, that this occurs via direct association of the *REST* gene and CHD2 protein, a process not mediated by H3K4me. This implies that cell stress caused by *CHD2* knockdown can be rescued by REST overexpression [109]. REST has been implicated as an important regulator of epileptogenesis following epilepsy-inciting insults, repressing the expression of critical neuronal genes like KCC2 and GRIN2A [122,123,124], however, the interaction between CHD2 and NRSF in this context, or indeed within the context of mature neurons has yet to be investigated.

#### 3.4.5. CHD2 Regulates Complex Tissue Development via Cell-Specific Mechanisms

Data from human embryonic stem cells (hESCs), showed that CHD2 is necessary for cellular differentiation. Indeed, CHD2 deficiency impaired the development of cINs from hESCs and altered electrophysiological characteristics of the cINs. This study further revealed that CHD2 regulates cIN development via interaction with the medial ganglionic eminence (MGE) associated transcription factor NKX2-1 [111].

*Chd2* heterozygous mice have reduced GABAergic progenitor proliferation compared to WT mice. Conversely, in glutamatergic populations transcriptional changes resulted in heightened synaptic activity [110]. Surprisingly, the authors noted that knockdown of Chd2 did not result in downregulation of REST in any of the assayed brain regions, contrary to the earlier findings of Shen and colleagues although age-related discrepancies may account for this [110].

## 4. Modeling CHD2-Opathies

### 4.1. Zebrafish Models of Chd2 Knockout

Pioneering work from the Baraban lab has demonstrated the suitability of Zebrafish, *Danio rerio*, for the study of developmental epileptic encephalopathies [125,126,127,128]. A Zebrafish *chd2* knockdown model was generated using targeted morpholino antisense oligomers [69]. Upon reduction in Chd2 levels the authors reported abnormal movements and seizure-like epileptiform discharges in mutant larvae as compared to their WT counterparts confirmed via field potential recordings [69].

Following this work, Galizia and co-authors used this Chd2 knockdown zebrafish and altered the field potential recording methodology to compare light and dark recording periods. *Chd2* knockdown zebrafish larvae displayed markedly increased photosensitivity (an abnormal cortical response to flickering light) as compared to WT controls [75]. Since photosensitivity is a common aspect of the epileptic phenotype in many CHD2 encephalopathy cases, the findings of these two *Chd2* knockdown zebrafish studies strongly support the implication of de novo *CHD2* mutations in epilepsy in humans.

### 4.2. Mouse Models of CHD2-Opathies

The first animal model of *Chd2* mutation was generated in mice in 2006 by Marfella and colleagues [24]. The authors generated homozygous and heterozygous CHD2 mutants via mouse ESCs harboring an insertion of a retroviral gene-trap at the *Chd2* locus. The induced mutation resulted in truncated CHD2 protein that lacked the C-terminal DNA binding domain. They demonstrated that homozygous mutants were non-viable, displaying perinatal lethality and developmental abnormalities prior to death. They also found that heterozygous mutation severely affects development, causes gross kidney abnormalities, and severely decreases longevity/survival [24].

Kulkarni and co-authors later reported the generation of another CHD2 deficient mouse line, also using a gene-trap system. In line with the findings of Marfella and colleagues they found that disturbances in *Chd2* led to embryonic and perinatal lethality. Further, they found that expression of *Chd2* is ubiquitous, but that regional expression of *Chd2* in cardiac, forebrain, facial and dorsal tissues was developmental stage specific in mouse embryos. The heterozygous (*Chd2^+/^*^-^) mice displayed growth retardation and morphological abnormalities including lordokyphosis, lowered body fat, and postnatal runting [101].

A *Chd2* heterozygous mutant mouse line was again generated by gene-trap cassette in 2009. The induced mutation was found to affect the region downstream of the DNA binding domain, causing disruption of the C-terminal end of the protein. The heterozygous mutants showed extramedullary haematopoiesis and increased susceptibility to lymphomas. The *Chd2^+/-^* mice displayed aberrant DNA damage repair and genomic integrity [57].

More recently, a heterozygous deletion (*Chd2^+/-^*) mouse line was generated by crossing transgenic mice containing loxP-flanked exon 3 of *Chd2* with a b-actin Cre line [110]. The generated mutant mice were found to produce around half as much CHD2 as their WT counterparts. These mice displayed altered neuronal development and neuron proliferation profiles, resulting in cognitive and long-term memory deficits [110]. Transcriptome analysis showed that *Chd2^+/-^* mice displayed divergent expression of a variety of genes responsible for chromatin regulation, neurogenesis, and synaptic transmission [110].

### 4.3. Induced Pluripotent Stem Cell (iPSC) Model of CHD2 Loss of Function

Although so much important information has been acquired by the use of the abovementioned *Chd2* animal models, there is still a need to develop and understand the effects of these mutations in human cells. Human induced pluripotent stem cell (hiPSC) lines modelling loss of function (LOF) of CHD2 have been developed. These lines were generated by combining the steps of somatic cell reprogramming to iPSCs and the induction of LOF mutations using CRISPR/Cas9 guide RNA (gRNA) delivered via episomal vectors [129]. The technique induces the LOF mutation in the gene of interest in the somatic precursors of the iPSCs prior to reprogramming. Thus, the generated iPSCs either carry the mutation (45%) or do not (55%), leading to the convenient acquisition of a pool of isogenic control cells [129]. The generation of these iPSC lines and isogenic controls is exceptionally useful in modeling the generality of LOF of CHD2 in human cells. Existing methods, including those used by Tidball and colleagues, exploit the generation of indels at the site of double stranded breaks [129,130]. Thus, there still exists the need to generate iPSCs from somatic cells of *CHD2* variant patients (or the development of a system for the efficient generation of specific mutations in iPSCs or their precursors) in order to investigate specific disease-causing mutations in vitro. Advances are rapidly being made in the field of directed mutagenesis, including highly efficient base editing systems [130,131] which will likely result in the ability to model any given patient mutation in the near future.

## 5. Therapeutic Opportunities/Future Perspectives

The molecular consequences of *CHD2* variants are, at present, difficult to predict. While some progress has been made, our understanding of how mutations in *CHD2* drives the development of hyperexcitable neuronal circuits is still poorly developed.

A more comprehensive understanding of how CHD2 regulates both neuronal development and brain function may provide key insights into the variable phenotype observed in patients with known *CHD2* mutations. There are many rapidly developing technologies which will, in time, undoubtedly facilitate the creation of human specific models of CHD2 variation. Most prominent of these, are the iPSC based models of the disease (including the development of brain organoids). When coupled to technologies involving directed mutagenesis using “base editing” or Crispr/Cas9 based approaches, such technologies promise to soon make accessible essential information relating to specific CHD2 variants. In addition, extant in silico methods like gene target network analysis and next generation sequencing (NGS) techniques and analysis tools, can be applied to the information that will be generated, providing the potential for delineating the complexity of regulatory systems governing CHD2-mediated transcriptional control.

Beyond this, such information may also inform novel treatment options for patients, who are often extremely drug refractory and have uncontrolled seizures, aggression and cognitive impairment. Once a definitive set of CHD2 targets has been identified and characterized, the modulation of a specific pathway or specific individual genes could be investigated for the alteration of expression of key proteins in the CNS phenotype. The possibility that the CHD2 “phenotype” is influenced by auxiliary molecules such as the regulatory lncRNA Chaserr must also be considered and investigated. Indeed, targeting of such molecules using antisense oligonucleotide approaches may offer the possibility to correct or enhance CHD2 expression and/or function [118].

Recent advances in these technologies should spur further research in these areas with the ultimate aim of mitigating the widespread effects of *CHD2* mutations.

## Figures and Tables

**Table 1:** Some human diseases associated with mutations in CHD family proteins.

CHD Family Mutation	Associated Pathology	Reference
CHD1	Prostate cancer and prostate cancer invasiveness	[65,67,68]
CHD2	Developmental Epileptic Encephalopathy	[69]
CHD3	Dermatomyositis, Hodgkin’s lymphoma Intellectual disability, macrocephaly, and impaired speech and language	[27] [70]
CHD4	Dermatomyositis, Sifrim–Hitz–Weiss syndrome	[71] [40]
CHD5	Neuroblastoma	[54]
CHD6	Colorectal cancer, Bladder carcinoma	[66] [13]
CHD7	CHARGE syndrome	[58]
CHD8	Autism spectrum disorder	[59]

**Table 2:** Early reported mutations in 15q20-15q26.1→qter (excluding ring chromosome 15) leading up to elucidation of a CHD2-DEE.

Mutation	Reference	Year	Variance Type	Phenotype
45,XY t(15;13)(15pter→15q26:13qll→13qter)	Pasquali et al. [Reported in [78,85]	1973	translocation	
del 15q21	[86]	1982	de novo deletion	
del 15q22–q24	[87]	1984	de novo deletion	
del 15q24→qter	[88]	1987	unbalanced translocation	
del 15q24→qter	[88]	1987	unbalanced translocation	
del 15q21 – q24	[89]	1988	de novo deletion	
15q22–q25	[89]	1988	de novo deletion	Intrauterine growth retardation (IUGR), hypertelorism, epicanthic folds, narrow, slanting palpebral fissures, and a short nose, abnormal insertion of the toes, craniofacial dysmorphic features, narrowing of the palpebral fissures, slight microphthalmia, strabismus, hypopigmentation of the irides, microcephaly (-5 SDS), metopic bulging, microretrognathia with a constantly opened mouth arched palate. Large ears with thick helixes and prominent tragi and antitragi
*15q26	[78]	1991	de novo deletion	IUGR, microcephaly, abnormal face and ears, micrognathia, highly arched palate, renal abnormalities, lung hypoplasia, failure to thrive, and developmental delay/mental retardation
15q26.1 [46,XXt(3;15)(q29;q26.1)]	Rosenberg et al. [Reported in [90]]	1992	balanced translocation	Left-sided diaphragmatic hernia, complex congenital heart disease. IUGR, low-set and posteriorly rotated ears, mild micrognathia, fifth fingers with only a single phalangeal crease, and bilateral single transverse palmar creases, Clinodactyly of the second and third toes, nails were hypoplastic on all digits, expired 20 min after birth
del 15q26.1→qter	[81]	1995	balanced translocation	IUGR, left diaphragmatic hernia, lung hypoplasia, cardiac dysmorphia, micrognathia, facial abnormalities
del 15q26.1→qter	[81]	1995	de novo deletion	IUGR, developmental delay, low set ears, relative, high arched palate, micrognathia,
15q25–15q26.2 [46,XX,del(15)(q25q26.2)]	[85]	2001	balanced translocation	IUGR, left-sided CDH with marked mediastinal shift and dextrocardia, left ventricular disproportion and talipes equinovarus (‘rocker-bottom feet’)
del 15q26.1	[91]	2001	de novo deletion	IUGR, cardiac defects, developmental delay, severe failure to thrive, micrognathia, low set ears, a broad nasal bridge, and a short neck
del 15q26.1	[92]	2003	de novo deletion	Hypoglycemia, cardiac abnormalities, moderate developmental delay
15q26.1	[93]	2004	de novo deletion	Diaphragmatic hernia, lung hypoplasia, cardiac dysmorphia, microretrognathia, hirsutism, facial abnormalities
del 15q26.1→qter	[94]	2005	de novo deletion	IUGR, lung hypoplasia, cardiac abnormalities, flat nasal bridge, a large anterior fontanelle, short palpebral fissures, bulbous nose, both ears with hypoplastic helices, redundant posterior neck skin, a grade II/VI systolic murmur best heard at the apex, slightly hyperpigmented labia majora with mild rugation, a deep sacral pit, clinodactyly of the 4th and 5th fingers of both hands, ulnar curvature of the 2nd finger of both hands, and medial deviation of all toes with a mild bilateral equinovarus, cholestatic liver disease, small kidneys, and spinal cord abnormalities
del15q26.2→qter	[95]	2005	de novo deletion	Seizures, failure to thrive, developmental delay, mild facial dysmorphia, proximal placed thumbs, terminal tapering digits, rocker bottom feet, severe growth retardation
del15q26.2	[96]	2007	de novo translocation (leading to 4.7 mb deletion)	developmental delay, growth retardation and some minor facial and limb dysmorphologies
del15q26.2→qter	[97]	2007	de novo deletion	Severe pre- and post-natal growth retardation, congenital heart malformation, facial asymmetry, oculocutaneous albinism without misrouting and subluxation of the radial heads
del15q26.2→qter	[98]	2008	de novo deletion	Severe growth retardation, microcephaly, and elevated IGF-I levels
del15q26.2→qter	[99]	2008	de novo deletion	Pre- and post- natal growth retardation, dysmorphic features including hypertelorism, facial abnormalities and rocker bottom feet, cardiac abnormalities, and severe cognitive disability, gastrooesophageal reflux.
15q26.1 [t(15;22)(q26.1;q11.2)]	[79]	2008	balanced translocation	Developmental delay, febrile seizures, left eye amblyopia, mild dysmorphic features including anteverted nares, unilateral auricular pit, fingertip pads, a low posterior hairline, and hirsutism at his back
del15q26.2→qter	Rump et al. (Reporting new data on probands first reported by Drayer et al. in 1977 [100]	2008	de novo deletion	Postnatal growth retardation, microcephaly, motor delay, excessive scalp hair, a broad nasal bridge, epicanthal folds, blepharophimosis, a right-sided divergent strabismus, low-set dysplastic ears with a narrow slit-like meatus, hyperextensible joints, and café au lait spots on her right cheek, hand and thigh, brachydactyly characterized by short second and fifth fingers, small thumbs and talipes equinovarus with short toes, absence of the middle phalanges of the second and fifth fingers, short middle phalanges of the third and fourth fingers, and short proximal phalanges of the thumb. The middle phalanges of the second to fifth toes were missing bilaterally, and the proximal phalanges of the first toes were short
del15q26.2→qter	Rump et al. (Reporting new data on probands first reported by Drayer et al. in 1977) [100]	2008	de novo deletion	Same as sister, but also including triangular facies and cardiac anomalies, hypospadia, cryptorchidism
15q26.1 [t(X;15)(p22.q2;q26.1)]	[101]	2008	balanced translocation	“Scoliosis, hirsutism, learning problems and developmental delay, high-arched palate, 2–3 syndactyly of the toes, and mildly elevated serum testosterone”
**15q26.1–26.2	[82]	2009	de novo deletion	“Refractory myoclonic epilepsy, mental retardation, growth delay, peculiar facial appearance, and minor physical anomalies”
***del15q26.2→qter	[102]	2011	de novo deletion	“Short stature and mental retardation, ventricular septal defect (VSD) which closed spontaneously at 6 years of age, no dysmorphic features”
***del 15q26.1	[83]	2011	de novo deletion	“Pervasive developmental disorder, growth delay, mild dysmorphic features, and intractable primary generalized epilepsy with a de novo microdeletion of approximately 0.73-0.94 Mb within chromosome 15q26.1”
***15q26.2–15q26.3	[103]	2011	de novo deletion	“Short stature and atypically mild developmental delay, thin vermilion of the upper lip, mild hypoplasia of the alae nasi, clinodactyly of the fifth fingers bilaterally, mild brachydactyly, short hands, short nails, both hands showed shortening of all metacarpals bilaterally, the metacarpals being of similar length to the proximal phalanges, hypoplasia of the fifth middle phalanges bilaterally”

* Roback et al. was the initial de novo mutation in the 15q26.1 region to describe the symptoms associated with the CHD2 EE phenotypes. ** The Veredice report is the first clearly defined case of CHD2 related myoclonic epilepsy. *** The three 2011 papers following the Veredice paper illustrate the role of CHD2 variance in the epilepsy component of the phenotype, the Dateki and Rudaks 15q26.2 variant patients (Dateki et al., 2011; Rudaks et al., 2011) display overlapping phenotypes but no epilepsy.
